# Management of the first stage of convulsive status epilepticus in adults: a systematic review of current randomised evidence

**DOI:** 10.1007/s00415-022-10979-2

**Published:** 2022-01-30

**Authors:** Moira Cruickshank, Mari Imamura, Carl Counsell, Lorna Aucott, Paul Manson, Corinne Booth, Graham Scotland, Miriam Brazzelli

**Affiliations:** 1 Health Services Research Unit, University of Aberdeen, Aberdeen, UK; 2 Institute of Applied Health Sciences, University of Aberdeen, Aberdeen, UK; 3 NHS Grampian, Aberdeen, UK; 4 Independent Consultant, Health Economist, Glasgow, UK; 5 Health Services Research Unit and Health Economics Research Unit, University of Aberdeen, 3rd Floor, Health Sciences Building, Foresterhill, Aberdeen, AB25 2ZD UK

**Keywords:** Convulsive status epilepticus, Benzodiazepines, Antiepileptic drugs, First-line treatment, Review

## Abstract

**Background:**

Convulsive status epilepticus is the most severe form of epilepsy and requires urgent treatment. We synthesised the current evidence on first-line treatments for controlling seizures in adults with convulsive status epilepticus before, or at, arrival at hospital.

**Methods:**

We conducted a systematic review of randomised controlled trials (RCTs) assessing antiepileptic drugs offered to adults as first-line treatments. Major electronic databases were searched.

**Results:**

Four RCTs (1234 adults) were included. None were conducted in the UK and none assessed the use of buccal or intranasal midazolam. Both intravenous lorazepam and intravenous diazepam administered by paramedics were more effective than placebo and, notably, intramuscular midazolam was non-inferior to intravenous lorazepam. Overall, median time to seizure cessation from drug administration varied from 2 to 15 min. Rates of respiratory depression among participants receiving active treatments ranged from 6.4 to 10.6%. Mortality ranged from 2 to 7.6% in active treatment groups and 6.2 to 15.5% in control groups.

**Conclusions:**

Intravenous and intramuscular benzodiazepines are safe and effective in this clinical context. Further research is needed to establish the most clinically and cost-effective first-line treatment and preferable mode of administration. Head-to-head trials comparing buccal versus intranasal midazolam versus rectal diazepam would provide useful information to inform the management of the first stage of convulsive status epilepticus in adults, especially when intravenous or intramuscular access is not feasible. Approaches to improve adherence to clinical guidelines on the use of currently available benzodiazepines for the first-line treatment of convulsive status epilepticus should also be considered.

## Introduction

Convulsive status epilepticus is the most severe form of epileptic attack and a life-threatening neurological emergency, which is associated with substantial mortality and morbidity [1–4]. The clinical manifestation of convulsive status epilepticus is characterised by a prolonged tonic–clonic seizure or repetitive seizures without full recovery of consciousness between them [2, 5, 6].

Epidemiological studies have documented a global annual incidence of status epilepticus of 7 to 41 cases per 100,000 population. In Europe, the annual incidence of status epilepticus lies between 10 and 16 per 100,000 population, and convulsive status epilepticus accounts for 45–74% of all cases [7–9]. Incidence of convulsive status epilepticus tends to be higher in males than females [4]. Mortality of status epilepticus has been reported to range from around 8–33% according to aetiology, with older age being a detrimental factor [7]. A recent meta-analysis of convulsive status epilepticus in high-income countries reported pooled mortality of 15.9% and the authors noted that survival rates have not improved over the last 30 years [10].

The ultimate goal of treatment is to stop both clinical and electroencephalographic seizure activity as soon as possible as convulsive status epilepticus can worsen with delayed or suboptimal treatment [5, 11–13]. Early treatment of convulsive status epilepticus is associated with reduced morbidity and mortality and with a greater proportion of terminated seizures at arrival at the hospital emergency department [14–16].

The first-line treatment of status epilepticus is currently benzodiazepines. The UK NICE Clinical Guidance recommends the use of buccal midazolam in the community setting before arrival at the hospital, or the administration of rectal diazepam if buccal midazolam is not available [17]. The Scottish SIGN guideline and the 2010 European Federation of Neurologists recommend intravenous (IV) administration of lorazepam or diazepam if IV access is already established and resuscitation available [12, 18]. To date, few trials have evaluated treatment options for adults and there is uncertainty about the optimal first-line treatment to control seizures before arrival at the hospital.

### Objectives

The objective of this assessment was to synthesise current evidence on first-line pharmacological interventions to control seizures in adults before, or at, arrival at the hospital with the aim to inform clinical practice and future research.

### Methods

We conducted a systematic review according to current methodological standards and pre-specified its methods in a research protocol (PROSPERO registration: CRD42020201953) (https://www.crd.york.ac.uk/prospero/display_record.php?RecordID=201953). This report adheres to the principles of the PRISMA 2020 statement [19].

### Information sources and search strategy

To identify eligible studies in the literature, we developed comprehensive search strategies and searched major electronic databases (Medline, Embase, and PsycInfo, EBSCO CINAHL, and Cochrane CENTRAL). Reference lists of included studies and websites of relevant professional organisations were checked for potentially eligible studies. All searches were conducted in July 2020, with no publication date or language restrictions. Details of the search strategies are reported in Appendix 1.

### Study selection

We included randomised controlled trials (RCTs) assessing pharmacological treatment versus placebo or active treatment for adults (≥ 16 years old) with convulsive status epilepticus. We focused on RCTs because, compared to any other study designs, they are more likely to provide unbiased information on the effects of pharmacological interventions for the treatment of convulsive status epilepticus in the adult population. Patients with a known epilepsy syndrome or with a reversible metabolic cause of seizures were deemed eligible for inclusion. Eligible interventions were any benzodiazepine regardless of their route of administration (e.g. intravenous (IV), intramuscular (IM), intranasal, buccal, rectal, or oromucosal) offered as first-line treatment for convulsive status epilepticus on site either by non-medical staff (i.e. caregiver) or paramedics, or at arrival at the hospital by emergency department staff. Newer antiepileptic drugs (AED) including levetiracetam, sodium valproate, and phenytoin were considered, so far as they were used as first-line treatment in the pre-hospital setting or at arrival at the emergency department. We considered first-line treatment as any immediate pharmacological treatment, which could be repeated once, and second-line treatment as any subsequent pharmacological treatment, which involved the use of another class of drug such as an anticonvulsant. Eligibility of participants was not restricted to a specific definition of status epilepticus. Traditionally, status epilepticus was defined as a seizure lasting 30 or more min, but more recent definitions indicate 5 or more minutes of either continuous seizure activity or repetitive seizures with no recovery of consciousness in between.

The main outcomes of interest were the following: seizure cessation (measured either in terms of number of people with cessation of seizure activity within 5–15 min of study drug administration [or any designated period as specified by trial investigators]; or time to seizure cessation from the time of study drug administration); recurrence of seizures (measured either as number of people with recurrence of seizures within a designated period, or time from seizure cessation to recurrence); and adverse events, namely respiratory depression and 30-day mortality.

### Data collection

Two review authors (MC, MI) independently screened all citations identified by the search strategies, retrieved and assessed for eligibility all potentially relevant full-text articles. The same review authors extracted data on study design, participants characteristics (number of participants in each group, demographic information), characteristics of intervention (provider, dose, and route of administration), and comparator intervention. The risk of bias of included trials was assessed by the same review authors using the revised Cochrane risk of bias tool for randomised trials (RoB 2) [20]. Each risk of bias domain was assessed separately for objective and subjective outcomes. For the risk-of-bias assessment, we categorised seizure cessation, recurrence of seizure, and respiratory depression (without ventilation) as subjective outcomes and mortality and respiratory depression (requiring ventilation) as objective outcomes.

At all stages of the study selection and data collection process, disagreement between reviewers was resolved by consensus or referred to a third review author (CC or MB).

### Data synthesis

We planned to conduct random-effects meta-analyses and subgroup analyses; however, due to the limited number of identified trials and their heterogeneity in terms of treatment comparisons and reported outcomes, this proved unfeasible. We also considered conducting a systematic review of economic evaluations but failed to identify sufficient evidence in the current literature. Results of each included study were tabulated and summarised narratively for each outcome.

## Results

### Study selection

**Fig. 1:**
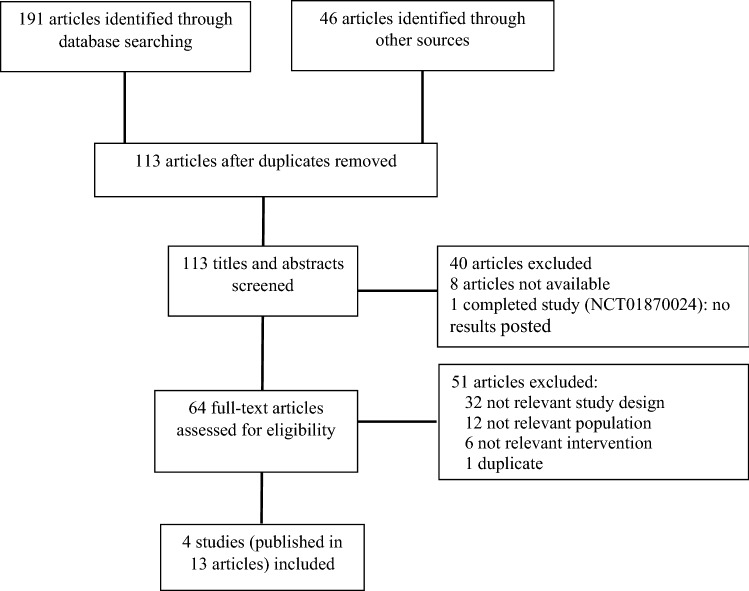
PRISMA flow diagram of study selection process

The literature searches identified 191 records. Forty-six additional records were identified from perusing the reference lists of selected studies and the websites of professional organisations. After assessing all potential relevant full-text articles in-depth, 13 articles reporting four studies met our inclusion criteria. Figure 1 presents the flow diagram of studies selection.

### Study characteristics

The study characteristics of the four included RCTs assessing a total of 1234 adults with convulsive status epilepticus are presented in Table 1 [15, 21–23]. The trials varied in size, ranging from 44 to 782 participants. Three trials were conducted in the USA[15, 22, 23] and one in France [21]. Three trials enrolled only adults[15, 21, 23] and the fourth trial included a mixed population of adults (89%) and children (11%) [22]. The RAMPART trial (Rapid Anticonvulsant Medication Prior to Arrival Trial) by Silbergleit et al. recruited adults and children with a bodyweight of at least 13 kg [22]. A publicly available dataset of participant-level data was obtained by contacting the authors and we were able to extract data for the 782 participants over 16 years of age. This assessment reports only the adults’ primary outcomes which were of clear origin, according to the accompanying data dictionary. Treatment comparisons of the trials were: IV lorazepam versus IV diazepam versus placebo [15]; IV levetiracetam plus clonazepam versus IV clonazepam [21]; phenobarbital plus phenytoin versus diazepam plus phenytoin [23]; and IM midazolam versus IV lorazepam [22]. The study by Shaner et al. was published in 1988 when the definition of status epilepticus and treatment regimen were likely to differ from the more recently published studies [23].

**Table 1:** Overview of study characteristics of the four included trials

Study ID	Country	Type of comparison	Study setting	Total number of participants randomised	Number of centres	Primary outcome
[15]	USA	2 mg IV lorazepam versus 5 mg IV diazepam versus IV placebo	Paramedics	205	10^a^	Termination of status epilepticus by arrival at the emergency department
[21]	France	2.5 g IV levetiracetam plus IV 1 mg clonazepam versus 1 mg IV clonazepam plus IV placebo	Paramedics	203	39^b^	Cessation of convulsions within 15 min of study drug administration
[23]	USA	100 mg/min IV phenobarbital plus 40 mg/min IV phenytoin versus 2 mg/min IV diazepam plus 40 mg/min IV phenytoin	Emergency department	44	1	Cumulative convulsion time
[22]	USA	10 mg IM midazolam versus 4 mg IV lorazepam	Paramedics	782	79^c^	Seizures terminated without need for rescue therapy before arrival at the emergency department

*IV* intravenous; *IM* intramuscular

^a^1 physician-staffed base hospital and 9 destination hospitals

^b^13 emergency medical service centres and 26 hospital departments

^c^4314 paramedics, 33 emergency medical centres and 79 receiving hospitals

### Participant characteristics

The mean age of participants in the four trials ranged from 48 years[22] to 55.9 years[23] in the active treatment arms and from 43.8 years[23] to 53 years[21] in the control groups. In all four trials, about half to three-quarters of the participants were male. In three of the included trials, the most common cause of convulsive status epilepticus was a sub-therapeutic level of antiepileptic drugs [15, 22, 23], while a brain lesion was reported as the most frequent cause in the fourth trial [21].

Table 2 presents a summary of the characteristics of participants in the four included trials.

**Table 2:** Summary of the demographic characteristics of the participants enrolled in the four included trials

Study ID	Study arm	*N* analysed	Age, years, mean (SD)	Gender (M/F), *n* (%)	Ethnicity, %	Final diagnosis, *n* (%)	Time from onset of convulsive SE to study drug admin; minutes, mean (SD) or median [range]
[15]	IV lorazepam	66	49.9 (20.1)	M 46 (70%) F 20 (30%)	Black: 18.2% White: 48.5% Other^a^: 33.3%	NR	34.0 (17.8)
	IV diazepam	68	50.4 (19.1)	M 41 (60%) F 27 (40%)	Black: 16.2% White: 54.4% Other^a^: 29.4%	NR	31.3 (14.5)
	IV placebo	71	52.0 (18.2)	M 42 (59%) F 29 (41%)	Black: 29.6% White: 46.5% Other^a^: 23.9%	NR	46.7 (38.8)
[21]	IV levetiracetam + clonazepam	68	55 (18)	M 49 (72%) F 19 (28%)	NR	SE: 66 (97.1%) Non-epileptic: 2 (2.9%)	58 [15–135]
	IV placebo + clonazepam	68	53 (18)	M 45 (66%) F 23 (34%)	NR	SE: 64 (94.1%) Non-epileptic: 4 (5.9%)	60 [20–258]
[23]	IV phenobarbital + phenytoin	18	55.9 (19.4)	M 13 (72%) F 5 (28%)	NR	GCSE*: 18 (100%) Other**: 0 (0%)	NR
	IV diazepam + phenytoin	18	43.8 (16.5)	M 9 (50%) F 9 (50%)	NR	GCSE*: 17 (94%) Other**: 1 (6%)	NR
[22]	IM midazolam	391	48 (17)	M 217 (56%) F 174 (44%)	Black: 54.0% White: 35.3% Other^a^: 10.7%	SE: 352 (90%) Non-epileptic: 28 (7%) Undetermined: 11 (3%)	NR
	IV lorazepam	391	49 (18)	M 203 (52%) F 188 (48%)	Black: 52.2% White 39.9% Other^a^: 7.9%	SE: 348 (89%) Non-epileptic: 29 (7%) Undetermined: 14 (4%)	NR

*GCSE* generalised convulsive status epileptics; *IM* intramuscular; *IV* intravenous; *NR* not reported; SD standard deviation; *SE* status epilepticus

^a^Other, mixed or unknown

*GCSE for entrance into study defined as a history of 30 min of continuous GCSE, and witnessed generalised seizures in the emergency room; or a history of 30 min of recurrent GCSE but failure to attain baseline mental status between seizures, and witnessed generalised seizures in the emergency room

**Includes a history of three or more GCSE in 1 h in patients with obtundation prior to the onset of status epilepticus and witnessed generalised convulsive seizures in the emergency room; or uncertain history of seizures but generalised convulsive seizures continuously for more than 5 min as witnessed in the emergency room

### Risk of bias assessment

The risk of bias assessments of individual trials are presented in Table 3. Three of the four trials were considered to have a low overall risk of bias [15, 21, 22], whilst the remaining trial (the smallest of the four trials) was judged to be at high risk of bias [23].

**Table 3:** Risk of bias of individual trials

Study ID	Intervention	Comparator	Outcome	Randomisation process	Deviations from intended interventions	Missing outcome data	Measurement of outcome	Selection of reported result	Overall
[15]	IV lorazepam	IV diazepam, IV placebo	Objective	+	+	+	+	+	+
[15]	IV lorazepam	IV diazepam, IV placebo	Subjective	+	+	+	+	+	+
[21]	IV levetiracetam + clonazepam	IV placebo + clonazepam	Objective	+	+	+	+	+	+
[21]	IV levetiracetam + clonazepam	IV placebo + clonazepam	Subjective	+	+	+	+	+	+
[23]	IV phenobarbital + phenytoin	IV diazepam + phenytoin	Objective	?	?	+	+	?	?
[23]	IV phenobarbital + phenytoin	IV diazepam + phenytoin	Subjective	?	?	+	−	?	−
[22]	IM midazolam	IV lorazepam	Objective	+	+	+	+	+	+
[22]	IM midazolam	IV lorazepam	Subjective	+	+	+	+	+	+

+ Low risk; ? Some concerns; − High risk; *IM*: intramuscular; *IV*: intravenous

### Results of individual trials

Table 4 presents a summary of the outcomes relating to seizure cessation and recurrence of seizure.

**Table 4:** Summary of clinical outcomes reported by the four included trials

Study ID	Arm	Seizure cessation			Recurrence of seizures	
		Number of people with cessation of seizure activity, *n* (%)	Effect estimate	Time to seizure cessation from admin of study drug, minutes	Number of people with recurrence of seizures, *n* (%)	Time from seizure cessation to recurrence, minutes, mean (SD)
[15]	IV lorazepam (*n* = 66)	39/66 (59.1%)	OR (95% CI)^a^ Lorazepam vs placebo: 4.8 (1.9, 13.0) Lorazepam vs diazepam: 1.9 (0.8, 4.4) Diazepam vs placebo: 2.3 (1.0–5.9)	HR (95% CI)^b^ Lorazepam vs placebo: 2.94 (1.41, 5.88) Lorazepam vs diazepam: 1.54 (0.85, 2.77)	NR	NR
	IV diazepam (*n* = 68)	29/68 (42.6%)			NR	NR
	IV placebo (*n* = 71)	15/71 (21.1%)			NR	NR
[21]	IV levetiracetam + clonazepam (*n* = 68)	50/68 (73.5%)	RR (95% CI) 0·88 (0·74–1·05)	Median 3 (range 0–50)	7/67 (10.4%)^c^	NR
	IV placebo + clonazepam (*n* = 68)	57/68 (83.8%)		Median 5 (range 0–41)	13/68 (19.1%)^c^	NR
[23]	IV phenobarbital + phenytoin (*n* = 18)	13/18 (72.2%)	NR	Median 5.5	NR	NR
	IV diazepam + phenytoin (*n* = 18)	6/18 (33.3%)		Median 15	NR	NR
[22]	IM midazolam (*n* = 391)	289/391 (73.9%)	NR	Median 3 (IQR 2, 6.3)	47/391 (12.0%)^d^	NR
	IV lorazepam (*n* = 391)	244/391 (62.4%)		Median 2 (IQR 1, 4.4)	42/391 (10.7%)^d^	NR

*AD* absolute difference; *HR* hazard ratio; *IM* intramuscular; *IV* intravenous; *NR* not reported; *OR* odds ratio; *RR* relative risk; *SD* standard deviation

^a^Adjusted for race or ethnic group, the intervals from the onset of status epilepticus to study treatment and from study treatment to arrival at the emergency department, and cause of status epilepticus within each prognostic group

^b^Adjusted for covariates (no further details provided)

^*c*^*p* = 0.16

^d^Within 12 h of ED arrival

Although definition of seizure cessation and of convulsive status epilepticus varied across the four included trials, our findings showed that, in general, benzodiazepines (i.e. lorazepam, diazepam, and midazolam) were effective at stopping seizures in adults treated in the pre-hospital setting. In the only trial with an untreated placebo arm by Alldredge et al., convulsive status epilepticus was successfully terminated before arrival at the emergency department in 59.1%, of adults treated with 2 mg IV lorazepam, in 42.6% of those treated with 5 mg IV diazepam and in 21.1% of those who received placebo, with no significant difference between the two benzodiazepine treatments (adjusted OR 1.9, 95% CI 0.8 to 4.4) [15]. The hazard ratio for the time between active treatment and seizure cessation was 2.94 (95% CI 1.41–5.88) for lorazepam versus placebo and 1.54 (95% CI 0.85–2.77) for lorazepam versus diazepam [15]. In the RAMPART trial by Silbergleit et al., 10 mg IM midazolam was non-inferior to 4 mg IV lorazepam for achieving seizure cessation (73.9% vs 62.4% of participants, respectively and the median time from active treatment to cessation of convulsions was similar across treatment groups: 2 versus 3 min in the midazolam and lorazepam groups, respectively) [22]. In the trial by Navarro et al., the addition of 2.5 g IV levetiracetam to 1 mg clonazepam did not confer any clear benefits over 1 mg clonazepam plus IV placebo in terms of the proportion of participants with seizure cessation (73.2% vs 83.8% participants, respectively) [21]. The median time between active treatment and interruption of the convulsion was 3 versus 5 min in the levetiracetam + clonazepam group and the clonazepam + placebo group, respectively [21]. In the study by Shaner et al., more participants treated with 100 mg/min IV phenobarbital plus 40 mg/min phenytoin achieved seizure cessation than those treated with 2 mg/min IV diazepam plus 40 mg/min phenytoin (72.2% participants vs 33.3%, respectively) [23]. The median time from active treatment to seizure cessation was shorter for the phenobarbital group than for the diazepam group (5.5 vs 15 min, respectively, *p* < 0.10) [23].

The number of participants with recurrence of seizures was reported by two trials and frequencies were similar between treatment arms of each individual trial. Navarro et al. reported that the proportion of participants who experienced recurrence of seizures during hospital stay was 10.4% in the levetiracetam plus clonazepam group and 19.1% in the placebo plus clonazepam group (RR 0·55, 95% CI 0·23 to 1·28, *p* = 0·16).[21] Silbergleit et al. recorded that 12% of participants in the IM midazolam group and 10.7% in the IV lorazepam group had recurrent seizures within 12 h after arrival at the emergency department [22].

Data on respiratory depression and mortality are shown in Table 5.

**Table 5:** Summary of safety outcomes reported by the four included trials

Study ID	Arm	Adverse events	
		Respiratory depression, *n* (%)	Mortality, *n* (%)
[15]	IV lorazepam (*n* = 66)	7/66 (10.6%)	5/65 (7.7%)
	IV diazepam (*n* = 68)	6/68 (8.8%)	3/67(4.5%)
	IV placebo (*n* = 71)	11/71 (15.5%)	11/70 (15.7%)
[21]	IV levetiracetam + clonazepam (*n* = 68)	7/68 (10.3%)^a^	3/66 (4.5%)^b^
	IV placebo + clonazepam (*n* = 68)	3/66 (4.5%)^a^	4/65 (6.2%)^b^
[23]	IV phenobarbital + phenytoin (*n* = 18)	NR	NR
	IV diazepam + phenytoin (*n* = 18)	NR	NR
[22]	IM midazolam (*n* = 514)^c^	33/514 (6.4%)	11/391 (2.8%)
	IV lorazepam (*n* = 509)^c^	51/509 (10%)	8/391 (2.0%)

*IM* intramuscular; *IV* intravenous; *NR* not reported

^a^*p* = 0.33

^b^*p* = 0.72

^c^Total enrolments

Respiratory depression was reported by three trials at low risk of bias and was generally low across the active treatment arms of individual trials, ranging from 6.4% for IM midazolam[22] to 10.6% for IV lorazepam [15]. In the Alldredge et al. trial, which included a placebo arm, respiratory depression was reported in 15.5% of participants who received placebo compared to 10.6% and 8.8% of those in the active treatment arms (lorazepam and diazepam, respectively) [15]. Mortality rates were higher in single placebo arms of individual trials but not significantly different between active treatment arms (see Table 4). Across trials, participants’ mortality ranged from 2.0% [22] to 7.6% [15] among participants who received IV lorazepam.

## Discussion

Current evidence from four RCTs (1234 adult participants in total) indicates that benzodiazepines are effective for the management of the first stage of convulsive status epilepticus in adults. All but one trial were judged at low risk of bias [23]. In general, evidence from the four trials shows that IV and IM benzodiazepines are safe and effective as first-line treatment in the pre-hospital setting compared with placebo. One trial evaluating the IV administration of lorazepam, diazepam, and placebo shows that seizure cessation is higher in the lorazepam group and the diazepam group compared with the placebo group but with no statistically significant differences between the two benzodiazepines. The RAMPART trial by Silbergleit et al., reports a higher rate of seizure cessation among people treated with IM midazolam than among those treated with IV lorazepam but the time from active treatment to seizure cessation is reported to be shorter with IV lorazepam (2 min) than with IM midazolam (3 min); however, the time from paramedic arrival to drug administration is not taken into account and appears to be longer in the IV group (4.8 min) than the IM group (1.2 min), reflecting the longer time needed to establish IV access [22]. When the total time from paramedic arrival to seizure cessation is taken into consideration, the difference between the two benzodiazepine groups is small.

The antiepileptic drug levetiracetam in combination with the benzodiazepine clonazepam appears to be safe but does not improve the rate of seizure cessation compared with clonazepam alone.

Overall, our findings are consistent with current clinical recommendations, which reflect the consensus of using benzodiazepines as the first-line treatment of convulsive status epilepticus [5, 11, 24]. It is worth noting, however, that despite the beneficial effects of benzodiazepines in the pre-hospital setting, a considerable proportion of participants who receive active treatment are still experiencing seizures on arrival at the hospital emergency department (from 16 to 67% of participants across trials).

Adverse events in terms of respiratory depression and mortality were generally low across trials with no statistically significant differences between treatment arms of individual trials.

A number of uncertainties have arisen from our findings. Buccal midazolam and rectal diazepam are currently recommended by NICE as first-line, pre-hospital treatment for people with prolonged or repeated seizures in the community as they can be administered immediately by trained carers in those at risk, without the need to wait for the paramedics to arrive. In the child population, buccal and intranasal midazolam have been reported to have similar efficacy for the early treatment of convulsive status epilepticus and the use of midazolam by non-IV route has been proposed as a favourable alternative to diazepam [25, 26]. We did not identify any trial in the literature assessing the use of buccal midazolam, rectal diazepam, or indeed intranasal midazolam, in the adult population. Head-to-head clinical trials comparing different benzodiazepines or different routes of administration would, therefore, be useful to inform clinical practice. In addition, it is currently unclear whether other doses of benzodiazepines than those used in the published trials would be effective and safe and future trials should consider addressing the question of optimal dosage of benzodiazepine use. Moreover, the appropriate level of training that paramedics should undertake to recognise and treat people with convulsive status epilepticus in the community has yet to be elucidated.

Further research is also needed to establish the cost-effectiveness of first-line treatments of convulsive status epilepticus. Future economic evaluations should aim at capturing the full cost of managing the convulsive epileptic episode to the time of discharge from the hospital.

### Strengths and limitations

This review was conducted following current methodological standards, including comprehensive literature searches of relevant sources and transparent methods throughout. In addition, we had access to the individual participant data for the largest trial. Limitations of the assessment include the identification of only a few published trials in the adult population, with small sample sizes and inadequate power to detect clinically important differences between active treatments. Differences across trials, in terms of the type of treatment administered and the choice and definition of outcome measures, hampered the possibility of conducting a meta-analysis.

## Conclusions

Current, limited evidence suggests that both 2 mg IV lorazepam and 5 mg IV diazepam administered by paramedics are more effective than placebo and 10 mg IM midazolam is non-inferior to 4 mg IV lorazepam. The addition of levetiracetam to clonazepam does not offer clear advantages over clonazepam alone. Large well-designed clinical trials are needed to establish which benzodiazepines are more effective and preferable for the first-line treatment of adults with convulsive status epilepticus. In particular, well-designed clinical trials in adults are needed to assess the use of IV lorazepam versus IV diazepam and to confirm the efficacy and safety of IM midazolam versus IV lorazepam. Future clinical trials comparing IM midazolam versus buccal or intranasal midazolam would provide useful information to inform the management of the first stage of convulsive status epilepticus in adults, especially when IV access is not feasible. Future cost-effectiveness analyses will also be useful to guide health policy and more cost-effective use of healthcare resources.

## Appendix 1 Clinical literature search strategies

Database: Embase < 1974 to 2020 Week 29 > , Ovid MEDLINE(R) and Epub Ahead of Print, In-Process & Other Non-Indexed Citations, Daily and Versions(R) < 1946 to July 17, 2020 > , APA PsycInfo < 1987 to July Week 2 2020 > 

1 Emergency Medical Services/use ppezv.

2 emergency health service/or emergency care/use oemez.

3 Emergency Services/use psyf.

4 (accident adj2 emergency).tw.

5 (“emergency room” or “emergency department” or ED).tw.

6 (pre-hospital or prehospital or “out of hospital” or community).tw.

7 Allied Health Personnel/use ppezv,psyf.

8 paramedical personnel/use oemez.

9 (paramedic* or ambulance).tw.

10 or/1–9.

11 Status Epilepticus/use ppezv, psyf.

12 epileptic state/use oemez.

13 Status Epilepticus.tw.

14 11 or 12 or 13.

15 exp Benzodiazepines/use ppezv,psyf.

16 exp benzodiazepine derivative/use oemez.

17 (midazolam or diazepam or lorazepam).tw.

18 exp Anticonvulsants/use ppezv.

19 exp anticonvulsive agent/use oemez.

20 exp Anticonvulsive Drugs/use psyf.

21 (levetiracetam or “sodium valproate” or phenytoin).tw.

22 or/15–21.

23 randomized controlled trial.pt. use ppezv.

24 controlled clinical trial.pt. use ppezv.

25 “randomized controlled trial”/use oemez.

26 “controlled clinical trial”/use oemez.

27 ((randomi#ed or controlled or clinical) adj2 (trial or study)).tw.

28 or/23–27.

29 10 and 14 and 22 and 28.

30 remove duplicates from 29.

## CINAHL

S1(MH “Emergency Medical Services”).

S2TX accident N2 emergency.

S3TX “emergency room” or “emergency department” or ED.

S4TX pre-hospital or prehospital or “out of hospital” or community .

S5(MH “Allied Health Personnel”).

S6TX paramedic* or ambulance.

S7(MH “Emergency Medical Technicians”).

S8 S1 OR S2 OR S3 OR S4 OR S5 OR S6 OR S7

S9(MH “Status Epilepticus + ”).

S10TX Status Epilepticus.

S11 S9 OR S10

S12(MH “Antianxiety Agents, Benzodiazepine + ”) .

S13TX midazolam or diazepam or lorazepam.

S14(MH “Anticonvulsants + ”).

S15TX levetiracetam or “sodium valproate” or phenytoin.

S16 S12 OR S13 OR S14 OR S15

S17TX (randomi#ed or controlled or clinical) N2 (trial or study).

S15 S8 AND S11 AND S16 AND S17

## CENTRAL

#1MeSH descriptor: [Emergency Medical Services] explode all trees.

#2(accident Near/2 emergency):ti, ab, kw (Word variations have been searched).

#3“emergency room” or “emergency department” or ED.

#4pre-hospital or prehospital or “out of hospital” or community.

#5MeSH descriptor: [Allied Health Personnel] explode all trees.

#6paramedic* or ambulance.

#7#1 or #2 or #3 or #4 or #5 or #6.

#8MeSH descriptor: [Status Epilepticus] explode all trees.

#9Status Epilepticus.

#10#8 or #9.

#11MeSH descriptor: [Benzodiazepines] explode all trees.

#12midazolam or diazepam or lorazepam.

#13#11 or #12.

#14#7 and #10 and #13.

#15MeSH descriptor: [Anticonvulsants] explode all trees.

#16(levetiracetam or “sodium valproate” or phenytoin):ti,ab,kw (Word variations have been searched).

#17#11 or #12 or #15 or #16.

#18#7 and #10 and #17.

## References

[1] Falco-Walter JJ, Bleck T (2016). Treatment of established status epilepticus. J Clin Med..

[2] Prasad M, Krishnan PR, Sequeira R, Al-Roomi K (2014). Anticonvulsant therapy for status epilepticus. Cochrane Database Syst Rev..

[3] Lesser RP, Johnson E. BMJ Best Practice: Status epilepticus. 2018. Available from: https://bestpractice-bmj-com.knowledge.idm.oclc.org/topics/en-gb/3000127. Accessed 5 October 2021

[4] Ascoli M, Ferlazzo E, Gasparini S, Mastroianni G, Citraro R, Roberti R (2021). Epidemiology and outcomes of status epilepticus. Int J Gen Med..

[5] Brophy GM, Bell R, Claassen J, Alldredge B, Bleck TP, Glauser T (2012). Guidelines for the evaluation and management of status epilepticus. Neurocrit Care.

[6] Fisher RS, Cross JH, French JA, Higurashi N, Hirsch E, Jansen FE (2017). Operational classification of seizure types by the International League Against Epilepsy: position paper of the ILAE commission for classification and terminology. Epilepsia.

[7] Logroscino G, Hesdorffer DC, Cascino G, Hauser WA, Coeytaux A, Galobardes B (2005). Mortality after a first episode of status epilepticus in the United States and Europe. Epilepsia.

[8] DeLorenzo RJ, Hauser WA, Towne AR, Boggs JG, Pellock JM, Penberthy L (1996). A prospective, population-based epidemiologic study of status epilepticus in Richmond, Virginia. Neurology.

[9] Betjemann JP, Lowenstein DH (2015). Status epilepticus in adults. Lancet Neurol.

[10] Neligan A, Noyce AJ, Gosavi TD, Shorvon SD, Köhler S, Walker MC (2019). Change in mortality of generalized convulsive status epilepticus in high-income countries over time: a systematic review and meta-analysis. JAMA Neurol.

[11] Glauser T, Shinnar S, Gloss D, Alldredge B, Arya R, Bainbridge J (2016). Evidence-based guideline: treatment of convulsive status epilepticus in children and adults: report of the Guideline Committee of the American Epilepsy Society. Epilepsy Curr..

[12] Scottish Intercollegiate Guidelines Network. Diagnosis and management of epilepsy in adults: SIGN 143. 2015. Available from: https://www.sign.ac.uk/sign-143-diagnosis-and-management-of-epilepsy-in-adults. Accessed 5 October 2021

[13] Walker M (2005). Status epilepticus: an evidence based guide. BMJ.

[14] Jagoda A, Riggio S (1993). Refractory status epilepticus in adults. Ann Emerg Med.

[15] Alldredge BK, Gelb AM, Isaacs SM, Corry MD, Allen F, Ulrich S (2001). A comparison of lorazepam, diazepam, and placebo for the treatment of out-of-hospital status epilepticus. N Engl J Med.

[16] Silbergleit R, Lowenstein D, Durkalski V, Conwit R, Investigators N (2013). Lessons from the RAMPART study—and which is the best route of administration of benzodiazepines in status epilepticus. Epilepsia.

[17] National Institute for Health and Care Excellence. Epilepsies: diagnosis and management [CG137]. 2012. Available from: https://www.nice.org.uk/guidance/cg137. Accessed 5 October 2021

[18] Meierkord H, Boon P, Engelsen B, Göcke K, Shorvon S, Tinuper P (2010). EFNS guideline on the management of status epilepticus in adults. Eur J Neurol.

[19] Page MJ, McKenzie JE, Bossuyt PM, Boutron I, Hoffmann TC, Mulrow CD (2021). The PRISMA 2020 statement: an updated guideline for reporting systematic reviews. PLoS Med.

[20] Sterne JAC, Savović J, Page MJ, Elbers RG, Blencowe NS, Boutron I (2019). RoB 2: a revised tool for assessing risk of bias in randomised trials. BMJ.

[21] Navarro V, Dagron C, Elie C, Lamhaut L, Demeret S, Urien S (2016). Prehospital treatment with levetiracetam plus clonazepam or placebo plus clonazepam in status epilepticus (SAMUKeppra): a randomised, double-blind, phase 3 trial. Lancet Neurol.

[22] Silbergleit R, Durkalski V, Lowenstein D, Conwit R, Pancioli A, Palesch Y (2012). Intramuscular versus intravenous therapy for prehospital status epilepticus. N Engl J Med.

[23] Shaner DM, McCurdy SA, Herring MO, Gabor AJ (1988). Treatment of status epilepticus: a prospective comparison of diazepam and phenytoin versus phenobarbital and optional phenytoin. Neurology.

[24] Shorvon S, Baulac M, Cross H, Trinka E, Walker M (2008). The drug treatment of status epilepticus in Europe: consensus document from a workshop at the first London Colloquium on Status Epilepticus. Epilepsia.

[25] Verrotti A, Ambrosi M, Pavone P, Striano P (2017). Pediatric status epilepticus: improved management with new drug therapies?. Expert Opin Pharmacother.

[26] McTague A, Martland T, Appleton R (2018). Drug management for acute tonic-clonic convulsions including convulsive status epilepticus in children. Cochrane Database Syst Rev..

